# Goalkeeper Game: A New Assessment Tool for Prediction of Gait Performance Under Complex Condition in People With Parkinson's Disease

**DOI:** 10.3389/fnagi.2020.00050

**Published:** 2020-03-04

**Authors:** Rafael B. Stern, Matheus Silva d'Alencar, Yanina L. Uscapi, Marco D. Gubitoso, Antonio C. Roque, André F. Helene, Maria Elisa Pimentel Piemonte

**Affiliations:** ^1^Department of Statistics, Federal University of São Carlos, São Carlos, Brazil; ^2^Department of Physical Therapy, Speech Therapy and Occupational Therapy, Faculty of Medical Science, University of São Paulo, São Paulo, Brazil; ^3^Department of Physiology, Institute of Biosciences, University of São Paulo, São Paulo, Brazil; ^4^Department of Computer Science, Institute of Mathematics and Statistics, University of São Paulo, São Paulo, Brazil; ^5^Department of Physics, School of Philosophy, Sciences and Letters of Ribeirão Preto, University of São Paulo, São Paulo, Brazil

**Keywords:** games 4 health, cognition, balance, gait, Parkinson's disease

## Abstract

**Background:** People with Parkinson's disease (PD) display poorer gait performance when walking under complex conditions than under simple conditions. Screening tests that evaluate gait performance changes under complex walking conditions may be valuable tools for early intervention, especially if allowing for massive data collection.

**Objectives:** To investigate the use of the Goalkeeper Game (GG) to predict impairment in gait performance under complex conditions in people with Parkinson's disease (PPD) and compare its predictive power with the one of the Montreal Cognitive Assessment (MoCA) test.

**Methods:** 74 PPD (HY stages: 23 in stage 1; 31 in stage 2; 20 in stage 3), without dementia (MoCA cut-off 21), tested in ON period with dopaminergic medication were submitted to single individual cognitive/motor evaluation sessions. MoCA and GG were used to assess cognition, and the dynamic gait index (DGI) test was used to assess gait performance under complex condition. GG test resulted in 9 measures extracted via a statistical model. The predictive power of the GG measures and the MoCA score with respect to gait performance, as assessed by DGI, were compared.

**Results:** The predictive models based on GG obtained a better score of prediction (65%) then MoCA (56%) for DGI scores (at a 50% specificity).

**Conclusion:** GG is a novel tool for noninvasive screening that showed a superior predictive power in assessing gait performance under complex condition in people with PD than the well-established MoCa test.

## Introduction

Parkinson Disease (PD) disabling is strongly related to gait impairments, decrease in independence in daily living activities and, consequently, reduction in Health-Related Quality of Life (HRQoL) (Morris et al., 2017).

Classically, gait was considered a mostly automatic motor skill. However, currently, gait control is regarded a complex brain process in which cognitive resources are continuously demanded in order to monitor the complex motor-perceptual integration (Yogev et al., 2005; Yogev-Seligmann et al., 2008; Amboni et al., 2013). Gait performance is strongly affected by the overlap between motor and cognitive impairments due to dysfunction of both dopaminergic and cholinergic pathways in PD (Kelly et al., 2015). Besides, low baseline CSF Aβ42 and, to a lesser extent, Aβ40 predicted gait alterations in the first 3 years following diagnosis, suggesting a role for amyloid pathology in gait-cognitive decline (Rochester et al., 2017). An association between axial signs and different aspects of cognition, particularly visuospatial learning and memory, was found in people at intermediate stages of PD (Schneider et al., 2015). Postural instability and gait disturbances were related to visuospatial function and visuospatial memory, while bradykinesia was associated with executive function in people at early stages of PD before intake of dopaminergic medication (Domellöf et al., 2011). Furthermore, gait predicted decline in specific cognitive domains (fluctuating attention and visual memory) in early PD, which was selective to discrete gait characteristic (Morris et al., 2017).

The person-environment model of mobility disability proposes that environmental demands can be categorized into eight dimensions: distance, temporal, ambient, terrain, physical load, attention, postural transitions, and density, representing the external demands required for an individual to be mobile within a particular environment (Patla and Shumway-Cook, 1998). Gait-related activities of daily living depend not only on the ability to walk at a minimum speed but also on the ability to adapt gait to diverse and complex task demands. Walking under complex conditions depend on the ability to modify and adjust gait to both expected and unexpected environmental challenges (Shumway-Cook et al., 2014).

Gait performance under complex conditions that involve high workload as walking over an obstacle (Stegemöller et al., 2012; Galna et al., 2013; Alcock et al., 2018), avoiding obstacles (Pieruccini-Faria et al., 2014), adapting gait to unexpected targets and obstacles (Caetano et al., 2018), is more affected in PD than gait performance under non-complex condition. Walking under these condition demands higher cognitive demands than unconstrained gait. Possibly, gait impairments in PD reflect altered motor control and overload of frontal networks, which magnitude is related to the underlying cognitive decline. People with PD and mild cognitive impairments display specific gait features as reduced step length and swing time and impairment of dynamic stability, which are only partially reversed by levodopa (Amboni et al., 2012). The higher activation in prefrontal cortex during obstacle negotiation walking in people with PD in comparison to elderly people confirmed the abnormal attentional demand during challenging walking conditions (Maidan et al., 2016). Moreover, people with PD were frequently attributed to a complex environmental condition such as challenging grounds, high attention demands, busy or cluttered areas and tasks requiring speed (Lamont et al., 2017).

New tools may provide sensitive methods of early non-invasive motor and cognitive screening in PD. Identification of early gait impairment could offer a critical opportunity for early intervention before gait changes with significant impact on independence in daily living activity, fall risk, and HRQoL.

Cognitive decline assessed by the Montreal Cognitive Assessment (MoCA) test has been associated with impaired ability to adapt the stepping behavior toward targets with obstacles (Caetano et al., 2018) and postural instability analysis (Pantall et al., 2018) in people with PD. MoCA scores were also associated with gait performance, freezing of gait, postural stability according to models adjusted for age, sex, education, enrollment site, disease duration, and motor symptoms severity (Kelly et al., 2015). The significant association between MoCA and step time variability is considered additional evidence of interplay between motor and cognitive networks (Rochester et al., 2017). These association may reflect less effective behavioral responses due to attentional control deficits and/or impaired cognitive function. In clinical routine, MoCA is widely used to evaluate the cognitive status in PD (Nasreddine et al., 2005; Gill et al., 2008; Hoops et al., 2009; Chou et al., 2010; Skorvanek et al., 2018), being able to detect alterations even in early stages of the disease (Kletzel et al., 2017). MoCA has also been considered a useful screening tool for PD global cognitive and executive functions (Vogel et al., 2015; Hendershott et al., 2017), even been applied to people with low educational level (Tumas et al., 2016).

The Goalkeeper Game (GG) has been recently introduced (de Castro, 2016) as a tool to investigate the conjecture that the brain does statistical model selection. Disturbance on gait performance and automaticity on PD are, both, related to dopaminergic loss. As GG is an instrument devoted to evaluate automaticity capacity it is a potential tool to indirectly evaluate dopaminergic loss and gait. GG is a videogame with internet, desktop and mobile device versions (http://game.numec.prp.usp.br) in which the player, taking the role of a goalkeeper in a soccer penalty shootout, guesses the position in the goal that the ball will hit (left side, right side or center). The game consists in a sequence of penalty kicks in which the ball positions can be generated either deterministically or randomly according to a strategy described by a tree and unknown to the player. The strategy is fixed for each phase and as the player (the goalkeeper) succeeds in guessing enough hits, which depends on the strategy tree, the phase terminates and a new one starts with a more complex tree. As the game evolves, the expectation is that for large numbers of trials in each phase the player is able to make sense of the strategy and obtain a high-scoring performance. Currently, the GG is being used by the Research, Innovation and Dissemination Center for Neuromathematics (http://neuromat.numec.prp.usp.br/) as an assessment tool in its basic and applied neuroscience researches.

GG allows for massive data collection, and it is expected that statistical analysis of the players' hit rates is sensitive to the cognitive decline associated to the players' decision-making models. Assuming that gait performance under complex conditions depends on the decision-making process underlying obstacle negotiation, speed selection, etc. (Yogev-Seligmann et al., 2008), it is plausible to suppose that GG performance is associated with gait performance under complex conditions (D'Alencar et al., 2018).

The purpose of this study was to investigate the predictive power of GG for impairments in gait performance under a complex condition in people with PD, and compare it with MoCA. The primary hypothesis of this study is that GG will achieve a gait performance prediction level similar to MoCA. Since GG allows for massive data collection, it could be used as a screening tool for gait impairments, enabling early intervention.

## Materials and Methods

### Participants

A convenient sample of 74 PPD recruited from the Laboratory of Motor Learning participated in this study. Inclusion criteria involved were individuals with (1) idiopathic Parkinson's disease as diagnosed by an experienced specialist in movement disorders, following the UK Brain Bank criteria (Hughes et al., 1992), taking antiparkinsonian medications, (2) in 1-3 disease stage according to Hoehn and Yahr scale (Hoehn and Yahr, 1967), (3) able to ambulate independently, (4) no signals of dementia (as determined by MoCA – cut-off 21) and/or major depression (as determined by Geriatric depression scale – cut-off 6). Subjects were excluded if they had clinically significant musculoskeletal, cardiovascular or respiratory disease, other neurological disease, or uncorrected visual/auditive disturbances.

### Design and Procedures

**Figure 1:**
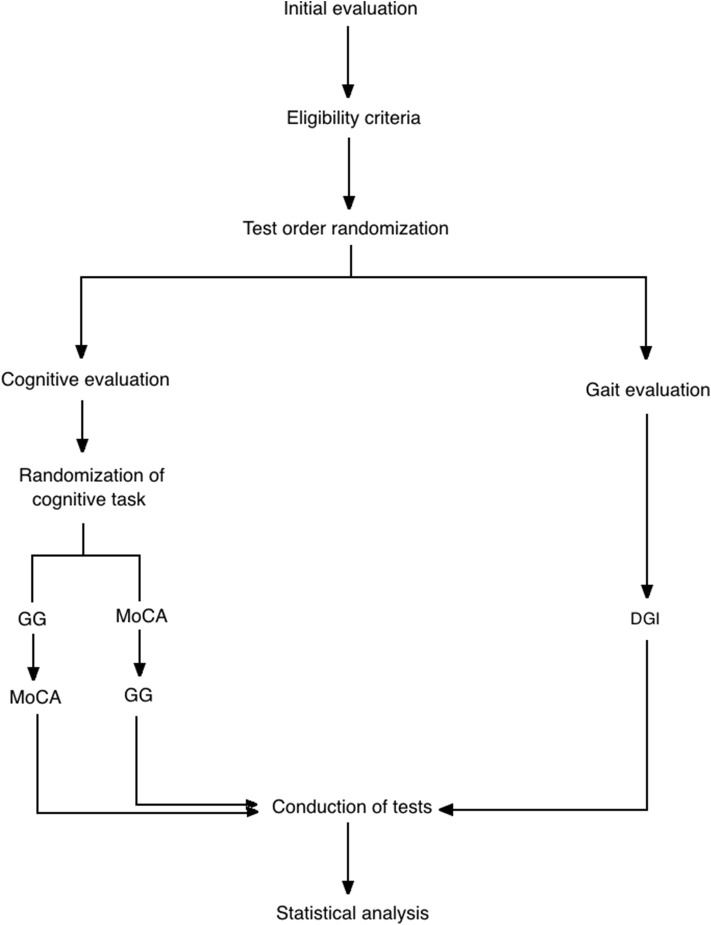
Flow diagram of the progress through the phases of study including enrollment, test order randomization (gait/cognitive test), cognitive test order randomization, assessment and data analysis. GG, goalkeeper game; MoCA, montreal cognitive assessment; DGI, dynamic gait index.

This study was approved by a Local Ethical Committee (# CAAE 67388816.2.0000.0065) and conducted in accordance with the Helsinki Declaration. A written informed consent was signed for each participant before the study begun. Based on a cross-sectional design, participants completed motor and cognitive evaluation in a single section. Evaluation order was randomized by sortition (Figure 1). Individual evaluation was conducted by a nurse and a physiotherapist specialized in movement disorders. All participants with PD were tested 40–120 min after their L-dopa dose (ON period).

### Cognitive Evaluation

Participants performed cognitive evaluation (GG and MoCA) seated comfortably in front of a desk where they could place elbows and forearms.

### GG Evaluation

GG was presented in a 23″ monitor (height = 29 cm, width = 51 cm) positioned 60 cm in front of the participants. After initial explanation about the game's rules, participants were asked to assume the goalkeeper role during the penalty shootout, pressing the selected key among three possibilities (◂, ▾ or ▸) which controls the direction of the goalkeeper's movement to defend the penalty (left, center, or right).

**Figure 2:**
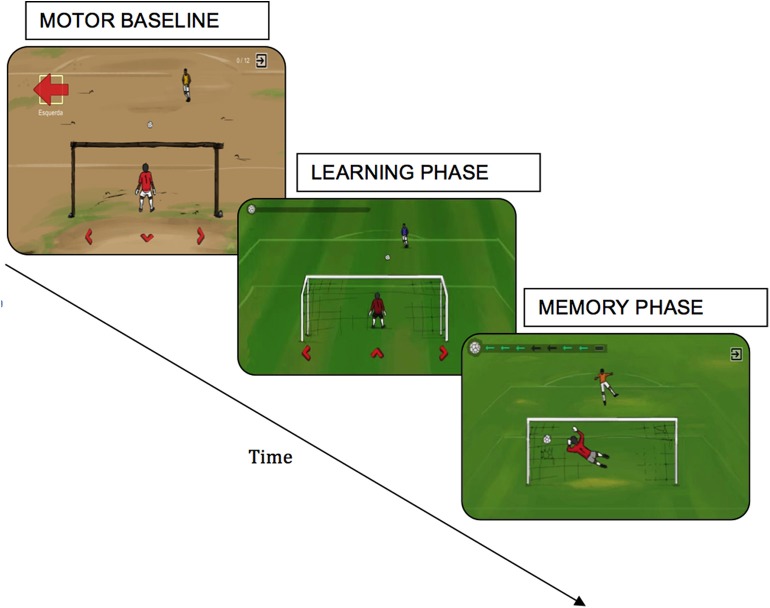
Goalkeeper Game where the participant was instructed to control the movements of a goalkeeper in time for a penalty, needing to guess the direction in which the opponent kicked the ball (left, right, or center). At each level, the opponent had adopted a new strategy based on implicit or explicit learning.

The GG version used in this study is a simplified one with only deterministic penalty sequences. It has 3 phases: (1) MOTOR BASELINE PHASE, in which visual cues were offered to participants to guide the correct direction (5 trials); (2) LEARNING PHASE, when no cues were offered and the sequences were provided by a deterministic model (20 trials); and (3) MEMORY PHASE, in which participants were asked to memorize the correct sequence before playing the game (5 trials) (Figure 2).

After every trial, participants received visual and auditory feedback indicating successful or unsuccessful attempt. All participants had to complete the three phases regardless of their performance. The entire session takes from 40 to 60 min.

### MOCA Evaluation

Motor evaluations were performed in a large room with adequate illumination and floor surface by a trained physiotherapist.

### Dynamic Gait Index (DGI)

This test was primarily designed to assess walking during challenging conditions. It includes both unconstrained gait and more complex walking tasks requiring the ability to modify and adapt gait to both expected and unexpected environmental conditions (Shumway-Cook et al., 2014). The DGI test evaluates not only usual steady-state walking but more complex abilities including walking while changing gait speed, walking while moving the head vertically and horizontally, walking while stepping over around an obstacle, pivoting during walking, and stair climbing. Performance is scored from 0 to 24 indicating, respectively, lowest and highest functioning level (Herman et al., 2009).

DGI is recommended by European PT guidelines for Parkinson's disease (Keus et al., 2013) as a test to assess gait performance. DGI has been demonstrated to have good retest, content, validity, construct, responsiveness and interrater reliability in PD. That reaches the “recommended” status for evaluation of gait and balance in PD (Bloem et al., 2016). Furthermore, it is considered useful as a supportive test for identifying fall risk in people with PD (Dibble et al., 2008).

After the initial explanation about the test, participants were asked to walk in habitual speed following the examiner's instructions.

### Statistical Analysis

GG variables cannot be compared directly to other clinical variables. For instance, for each stage and patient, GG consists of a sequence of failures/successes in the patient's predictions for that stage. Since each particular prediction carries little information, it is weakly correlated to the patient's clinical variables. In order to overcome this problem, we built a model that extracts each patient's overall GG performance. This model is a generalization of logistic regression (Friedman et al., 2001, p. 119). Specifically, for each time iteration, *t*, patient, *p*, and GG stage, *s*, we define *X*_*t, p, s*_ as the indicator that *p* made the correct prediction at iteration *t* of stage *s* of GG. The stages 1, 2, and 3 refer to, respectively, the motor baseline, learning and memory phases of the GG. The distribution of *X*_*t, p, s*_ is given by Equation (1):

(1) $$\begin{array}{l} \;\lambda_{t,p,s}=\left(t-1\right)\beta_{p,s}-\log\left(3\gamma_{p,s}-1\right)\\ \mathbb{P}\left(X_{t,p,s}=1\right)=\gamma_{p,s}\frac{\exp\left(\lambda_{t,p,s}\right)}{1+\exp\left(\lambda_{t,p,s}\right)} \end{array}$$

The model in equation 1 admits an intuitive interpretation. First, it is chosen so that the probability that a patient makes a correct prediction at the first iteration of each stage is 1. This is reasonable since, at that point, the patient has no information, and there are 3 options. Also, the parameter γ_*p,s*_ represents patient *p*'s total learning at stage *s*. That is, γ_*p,s*_ is the probability that *p* makes a correct prediction at *s* after playing that stage for a large number of iterations. Finally, β_*p,s*_ is the rate of learning of patient *p* at stage *s*. That is, β_*p,s*_ determines how many iterations *p* requires at stage *s* so that the probability of making a correct prediction is close to γ_*p,s*_.

Posterior estimates for β_*p,s*_ and γ_*p,s*_ were obtained using Stan^51^ (Carpenter et al., 2017). As a result, 3 pairs of β and γ were attributed to each patient. By complementing these values with the average time spent per iteration in each stage, we obtained 9 variables that measure the patient's GG performance.

The GG variables and the MoCA score are compared with respect to two criteria. Fist, pairwise correlations and linear models for DGI are contrasted. Second, the GG variables and MoCA are also compared with respect to predictive power for DGI. Specifically, we adjust classifiers based on either the GG or the MoCA score to predict whether DGI scores where above or below the median. The classifiers were adjusted using elastic-net regularized logistic regression (Zou and Hastie, 2005; Friedman et al., 2010).

All of the relevant code and data is available at https://github.com/rbstern/gg_analysis.

## Results

Demographic and clinical features of participants are shown in Table 1. Means and standard deviations of the estimated values of the 9 GG performance variables for these participants are given in Table 2.

**Table 1:** Demographic and clinical assessment data of the patients with Parkinson's disease (*n* = 74).

	**HY 1 (*n* = 23)**	**HY 2 (*n* = 31)**	**HY 3 (*n* = 20)**
Age (years)	68.87 (9.39)	63.55 (8.14)	69.95 (8.67)
Gender (male)	M (14)	M (22)	M (17)
Education (years)	11.30 (4.49)	12.90 (5.31)	15.40 (4.21)
L-Dopa (mg/day)	284.78 (190.36)	324.19 (169.25)	415.00 (215.88)
FoG-Q	3.13 (3.72)	5.29 (3.67)	8.10 (6.19)
UPDRS-III	14.42 (7.35)	19.71 (7.06)	25.95 (11.34)
MoCA (total)	24.78 (2.70)	24.90 (3.43)	24.25 (2.77)
Visuospatial/executive	3.78 (1.13)	3.58 (1.09)	3.35 (1.46)
Naming	2.65 (0.65)	2.77 (0.43)	2.85 (0.37)
Attention	5.13 (0.97)	5.03 (0.91)	4.80 (0.95)
Language	1.70 (0.88)	1.94 (0.96)	1.90 (0.91)
Abstraction	1.87 (0.34)	1.84 (0.45)	1.80 (0.62)
Memory (delayed recall)	3.43 (1.16)	3.55 (1.06)	3.15 (0.93)
Orientation	5.57 (0.59)	5.61 (0.80)	5.60 (0.60)
GDS	4.74 (3.00)	4.06 (2.53)	5.85 (3.23)

*For continuous variables, the mean value is presented and also the standard deviation in parenthesis. For categorical variables, the mode is presented and also the mode's proportion in parenthesis. GDS stands for the geriatric depression scale*.

**Table 2:** Means and standard deviations among patients of the estimated parameters for performance in the GG.

	**β_1_**	**β_2_**	**β_3_**	**γ_1_**	**γ_2_**	**γ_3_**	***t*_1_**	***t*_2_**	***t*_3_**
Mean	1.74	1.76	2.00	0.68	0.64	0.78	4.47	3.28	2.52
s.d.	0.53	0.38	0.37	0.16	0.14	0.18	6.21	3.78	2.40

As a first analysis, the MoCA and GG variables were tested using pairwise correlations and linear regressions for DGI. The polychoric and polyserial correlations (Drasgow, 2004) for each pairwise comparison are summarized in Table 3. The total learning in the memory phase of GG, γ_3_, is the variable that is most strongly correlated to DGI. Also, MoCA, γ_1_, *t*_1_, and *t*_3_ are moderately correlated with DGI. Similarly, in a proportional odds model (McCullagh, 1980) for DGI based on MoCA, the regression coefficient for MoCA is estimated as 0.11, and associated *p*-value of 0.10. Also, the estimates for the proportional odds model for DGI based on the GG variables are summarized in Table 4. Both the pairwise correlations and the linear model indicate that DGI is strongly associated to the memory stage of the GG but only moderately associated to MoCA.

**Table 3:** Pairwise polychoric and polyserial correlations between explanatory variables (MoCA and GG variables) and DGI.

		**Motor baseline**			**Learning phase**			**Memory phase**		
**Variable**	**MoCA**	**β_1_**	**γ_1_**	***t*_1_**	**β_2_**	**γ_2_**	***t*_2_**	**β_3_**	**γ_3_**	***t*_3_**
Correlation	0.20	−0.01	0.24	−0.23	−0.13	−0.06	−0.21	−0.12	0.39	−0.24
*p*-value	0.14	0.98	0.06	0.09	0.32	0.62	0.11	0.36	0.001^*^	0.07

*Also shown, the p-value for the hypothesis that each populational correlation equals 0*.

* *p < 0.05*.

**Table 4:** Estimates of the coefficients in a proportional odds model for DGI based on the GG variables.

	**Motor baseline**			**Learning phase**			**Memory phase**		
**Variable**	**β_1_**	**γ_1_**	***t*_1_**	**β_2_**	**γ_2_**	***t*_2_**	**β_3_**	**γ_3_**	***t*_3_**
**Coefficient**	−0.43	3.96	−0.03	−0.25	−3.60	−0.005	−2.57	5.28	−0.11
*p-value*	0.40	0.03	0.75	0.73	0.06	0.97	0.007^*^	0.003^*^	0.61

*Also shown, the p-value for the hypothesis that each coefficient is equal to 0*.

* *p < 0.05*.

**Figure 3:**
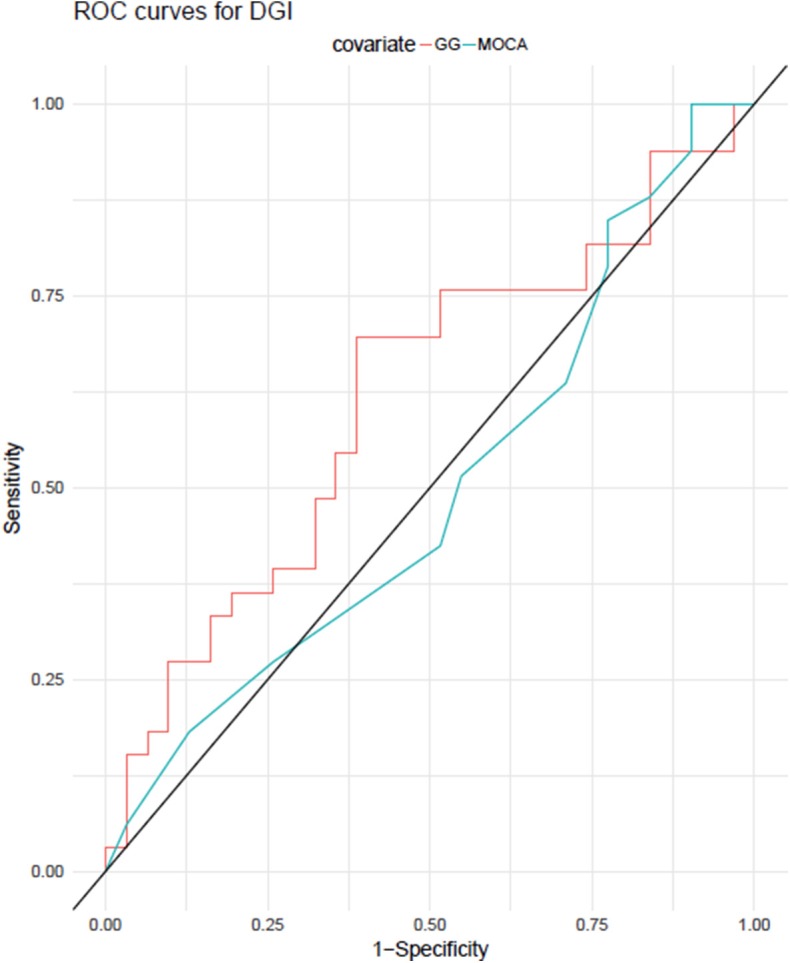
ROC (receiver operator characteristic) curves for predicting DGI using the classifiers adjusted with either GG variables or MoCA score.

The GG variables and MoCA were also used to classify whether DGI was below or above the median. Their cross-validated accuracies were, respectively, 65% and 54%. When a binomial test is used to determine whether a coin flip is as good as the classifier, the respective *p*-values are 0.019 and 0.54. This supports that, while GG has a moderate predictive power for DGI, MoCA does not. Also, Figure 3 presents the cross-validated receiver operating characteristic (ROC) curves for the classifiers based on GG and on MoCA. The areas under the curve (AUC) for each covariate were, respectively, 0.65 and 0.507. DeLong's test (DeLong et al., 1988) for the hypothesis that the area under the curve for GG is smaller or equal than that for MoCA yields a *p*-value of 0.084. This result might be explained by the fact that the difference in predictive power between the GG variables and MoCA for DGI is only moderate. A nonparametric test such as DeLong's cannot detect this difference for the available sample size.

## Discussion

The main finding of this study is that GG had a slightly higher predictive power for decline in gait performance under complex situations than MoCA. That corroborate with the reasoning that disturbance on gait performance and automaticity could be, both, consequences of PD dopaminergic loss.

Gait performance regard a complex process demanding to monitor complex motor-perceptual integration and, under complex conditions, depends on the interplay between cognitive and motor process. A disruption in this ability increases the risk of fall, decreases the independence in daily living activities and, consequently, decreases the HRQoL. The early detection of the decline in gait performance may open a novel therapeutics windows to prevent its progression. In this way, free and friendly tools able to broaden the screening capabilities for this kind of disruption in the population may offer significant improvements in comparison to existing tests. The result of the study presented here suggests that the GG, a game based on the most popular sport in the world (soccer), may be a possible candidate for such a screening test.

DGI was designed to measure the overall ability to engage in complex walking tasks, which require high dynamic balance (Shumway-Cook et al., 2014). This test was adopted in the present study to assess gait control under challenging gait conditions where the cognitive resources are more demanding than only walking. Previous studies showed higher activation in prefrontal cortex during obstacle negotiation in comparison to no-obstacle situation in the gait in young or older adults (Mirelman et al., 2017) and people with PD (Maidan et al., 2016). This evidence corroborates the assumption that more complex gait conditions demand more cognitive resources. Therefore, complex gait are more sensitive to assess the interplay between cognitive and motor process involved in gait control. Besides, obstacle negotiation tested via DGI is frequently performed in daily life and the performance in this test is associated with fall risk (Dibble et al., 2008; Bloem et al., 2016).

Based on this evidence, it is plausible to suppose that GG would be able to predict the level of impairment and fall risk in daily living activity in PPD. Further studies should be conducted to answer this research question.

The GG version developed for this study was designed with 3 phases, each of them designed to emphasize a motor or cognitive component (though all components were required during the whole game). The first phase was designed to predominantly evaluate motor performance. In this phase the cognitive demand was minimized by the indication of the correct shooting direction that should be selected by the player, i.e., no decision making was required. The second phase was designed to evaluate the ability to identify the correct sequence of shooting directions without cues. In this phase, the cognitive demand was higher than in the others because the decision-making process to select the next direction strongly depends on the executive function. Finally, the third phase was designed to evaluate attention and memory, including working memory. In this phase the player must memorize the sequence of shooting directions presented and, only after successful memorization, the game started. Hence, the main cognitive abilities required by the version of GG used in this study were attention and memory. The statistical model adopted to estimate GG performance allowed to classify the level of efficiency of the selected strategy to reach best results in each phase of the game.

A previous study showed that executive function deficits were associated with impairments in gait and postural stability (Rochester et al., 2017). Specifically, in fully adjusted models, deficits in executive function (processing speed, visuospatial function and working memory) were associated with severe gait impairments while memory deficits were associated with severe postural instability (Kelly et al., 2015). Moreover, dynamic stability was strongly associated with visuospatial impairment (Amboni et al., 2012). Among the global cognitive abilities required by GG, visuospatial attention, working memory and processing speed are crucial. Thus, it is not surprising that GG has reached a slightly superior predictive power than MoCA for gait performance under complex conditions. These conditions pose many balance challenges and demand good postural stability, confirming the central hypothesis presented in this study.

In particular, the performance in the third phase of GG depends on both declarative and working memory, as the participants must be able to memorize the correct sequence manage it using the working memory to select the correct direction. Thus, we explain the high correlation between the memory stage of GG and DGI scores because DGI assesses gait performance under balance-challenging condition. Further support for our finding of bidirectional association between declarative memory and gait performance under complex condition comes from the dual-task gait cost, another measure for gait performance under challenging condition, which predicts impaired performance in delayed recall in people with mild cognitive impairment, even after adjusting for the executive dysfunction (Montero-Odasso et al., 2014).

However, why should the clinicians and researchers adopt the GG as a potential marker that could increase the likelihood of early detection or prediction of gait impairments instead of MoCa?

Currently, it has been proposed that early PD could be divided into 3 stages: (1) preclinical, in which the neurodegenerative process is starting and there are no evident symptoms or signs of the disease; (2) prodromal, in which symptoms and signs of PD are present but are insufficient to define a full clinical picture; and (3) clinical, in which the diagnosis is achieved, based on the presence of classical motor signs. Finding new criteria to identify the prodromal phase represents an important challenge for PD research. A growing body of evidence has shown that gait control is already affected in the prodromal phases of Alzheimer disease and PD (Postuma et al., 2012; Montero-Odasso et al., 2014), and maybe a powerful prodromal marker of neurodegenerative diseases, including PD (Belghali et al., 2017a,b). Cognitive impairment is associated with gait alterations regardless of HY stage or UPDRS motor score, suggesting that cognitive impairment may serve as a proxy marker of gait disturbance or fall in PD (Kim et al., 2018).

Moreover, the progression of discrete gait alterations has been indicated as a promising clinical marker for cognitive decline (Amboni et al., 2013; Morris et al., 2017) and pathology and disease progression (Rochester et al., 2017). In this context, the main advantage of GG in comparison to current cognitive assessment tools is its potential for massive data collection. Considering the PD prevalence (1% in the general population), the use of cognitive and motor assessment instruments for an early screening of gait alterations is expensive and not feasible for most countries. Hence, a free and friendly game like GG, which allows for massive data collection and is able to detect early abnormal decline in gait performance may be extremely useful. Further transversal and longitudinal studies are certainly needed to validate the GG as a potential marker for early prediction of gait impairments in PD. The results of the present study should be considered an essential first step in this direction.

Finally, it is fundamental that we point some limitations of the present study for result generalizations. Small sample size and the absence of a control group may be considered the main limitations. Future studies must overcome these limitations.

## Data Availability Statement

The raw data supporting the conclusions of this article will be made available by the authors, without undue reservation, to any qualified researcher. The datasets generated for this study can be found in the https://github.com/rbstern/gg_analysis.

## Ethics Statement

This study was approved by a Local Ethical Committee (number CAAE 67388816.2.0000.0065) and conducted in accordance with the Helsinki Declaration. A written informed consent was signed for each participant before the study begun.

## Author Contributions

RS, Md'A, YU, MG, AR, AH, and MP contributed to the study concept and design. Md'A, YU, and MP collected and analyzed the data. RS performed the statistical analyses. RS, Md'A, MG, AR, AH, and MP contributed to the interpretation of the results. RS, Md'A, AR, AH, and MP drafted and revised the manuscript. All authors contributed to the critical revision of the manuscript. All authors approve the final manuscript as submitted and agree to be accountable for all aspects of the work.

### Conflict of Interest

The authors declare that the research was conducted in the absence of any commercial or financial relationships that could be construed as a potential conflict of interest.
